# The rehabilitation experiences, individual and combined effects of cognitive and physical rehabilitation on health and social outcomes in older athletes: A scoping review protocol

**DOI:** 10.1371/journal.pone.0343744

**Published:** 2026-03-06

**Authors:** Alexander Wakim, Kelvin Somoto Utazi, Nelson E. Ekechukwu, Michael E. Kalu

**Affiliations:** 1 School of Kinesiology and Health Science, York University, Ontario Canada; 2 Department of Medical Rehabilitation, Faculty of Health Science and Technology, University of Nigeria, Enugu Campus; 3 Emerging Researchers and Professionals in Aging – African Network, Toronto Canada; Portugal Football School, Portuguese Football Federation, PORTUGAL

## Abstract

**Background:**

Evidence indicates that combined cognitive and physical rehabilitation can yield substantial improvements in health and social outcomes within the general aging population. However, the specific effects of such interventions on older athletes, who often exhibit enhanced resilience due to their competitive training backgrounds, remain inadequately explored.

**Objectives:**

This study aims to describe the treatment regime (frequency, intensity, time/duration, and type of volume and progression of rehab) and components of cognitive and physical rehabilitation interventions, describe their combined effects on health and social outcomes and explore older athletes’ experiences with these interventions.

**Methods:**

We will conduct a systematic scoping review following the five stages of the Arksey and O'Malley Framework. The search strategy will be developed in collaboration with a health and rehabilitation librarian, and searched in multiple data bases: MedLine, Web of Science, ProQuest, EBM Reviews, JBI EBP, Embase, APA PsychInfo, and Social Services Abstracts. Multiple reviewers will screen titles and abstracts, followed by full-text assessments in COVIDENCE, utilizing predefined inclusion and exclusion criteria. Articles will be included if participants were older athletes who actively engage in organized sports, characterized by regular competition and systematic training, and who have undergone cognitive and physical rehabilitation interventions. Data extraction will occur in pairs, focusing on relevant information to fulfill the study objectives. The pilot testing of all components—titles, abstracts, full texts, and data extraction protocols—will be conducted prior to the main review process. We will categories the physical and cognitive interventions using as frequency, intensity, time/duration, type of volume, progression of rehabilitation, and type of exercise. Additionally, data on intervention effects, including effect size and mean differences, will be summarized narratively. The experiences of the older athletes would be thematically analyzed.

**Discussion:**

This scoping review will provide valuable information for clinicians, researchers, and policymakers to develop combined physical and cognitive rehabilitation programs tailored to the distinct physiological, cognitive and social needs of older athletes.

## Introduction

An athlete is defined as someone who meets several specific criteria including, actively training in sports to enhance performance, participating in sports competitions, registered with a sports federation at the local, regional, or national level, and prioritize sports training and competition as their main activity or interest [1]. This commitment often requires dedicating more time to these activities than to other professional or leisure pursuits. While athletes are typically younger individuals, global aging trends are driving a significant demographic shift in this group. As a result, many athletes continue their involvement in sports well into their older years, enjoying numerous health benefits including improved cardiovascular health, a lowered risk of chronic diseases, and better mental well-being [2–4].

While the older adult’s definition is relatively stable ranging from individuals aged 55 and above in the black population [5], to 60 and older [6] or 65 and older [7], the older athlete's definition varies by sport, reflecting diverse age-based classifications across athletic disciplines. The International Tennis Federation, for example, categorizes players into “young seniors” (ages 35–45), “seniors” (ages 50–60), and “super-seniors” (ages 65–85 for men and 60–80 for women) [8]. Other sports, such as track, and field consider those 35 + years as master’s athlete [9]. This cut-off is also consistent with physiological evidence showing that key performance indicators (e.g., sprint speed, power, and VO ~ 2max) typically peak in the 20s to early 30s and begin a gradual decline from the mid-30s [10]. Moreover, the 35 + definition is widely applied across regions and organizations (e.g., European Veterans Athletics Championships; Swedish Veteran Athletics) and aligns with the standard 5-year Masters age-band classification [11,12]. Building on these variations regarding an older athlete, we define an “older athlete” comprehensively to include all different definition with the lower age range. Therefore, an older athlete in our study, is an individual aged 35 or above who actively participates in an organized sport that emphasizes regular competition and systematic training. Because of the natural decline in bodily resilience and cognitive functions that comes with aging, older athletes may require physical/and/or cognitive rehabilitation.

Physical rehabilitation is defined as the use of health-based approaches to optimize physical function and participation in persons with physical impairments (e.g., mobility) or symptoms (e.g., low back pain) that are amenable to recovery, prevention, or management from physical rehabilitation services, facilities or providers (e.g., physical or occupational therapists) [13]. There is reputable high-quality evidence on the benefits of physical rehabilitation on several outcomes in the aging population including activities of daily living [14], improves strength, flexibility, balance, muscle strength, as well as help prevent injuries [15]. However, synthesized evidence on the role of physical rehabilitation on health and social outcomes among older athletes is lacking, highlighting the need for this study.

Cognitive rehabilitation is a structured, functionally driven therapeutic approach designed to address brain-behavioral deficits, based on a thorough assessment of the patient’s condition [16]. Various intervention strategies have been employed in the literature [17], including (1) reinforcing and restoring previously acquired behavioral patterns, (2) developing new cognitive processes through compensatory mechanisms to support impaired neurological functions, (3) implementing external aids such as assistive devices or environmental modifications to enhance cognitive function, and (4) assisting individuals in adapting to cognitive limitations when direct restoration or compensation is not possible, ultimately improving overall functionality and quality of life [16]. Areas of cognition that may be targeted include attention, concentration, perception, memory, comprehension, communication, reasoning, problem-solving, judgment, initiation, planning, self-regulation, and awareness [17]. Regardless of the specific approach or area of intervention, cognitive rehabilitation services should focus on achieving changes that enhance each person’s function in areas that are relevant to their daily lives; and would have significant benefit for older athletes. And yet, there remains a lack of synthesized evidence on the role of cognitive rehabilitation in improving health and social outcomes for older athletes.

Cognitive motor training (CMT) is defined as the simultaneous execution of a cognitive and a physical task [18]. Extensive research on CMT does exist [18–23] but it is focused mostly on older adults and not on older athletes with buildup resilience as a result of competitive training. These studies have demonstrated that CMT in older adults is effective in yielding significant improvements in both cognitive and motor skills compared to either type of training alone [18–21,23]. Consequently, integrated training programs that incorporate dual-tasking (DT) or multitasking have been developed to enhance the efficacy of cognitive and motor training and to improve performance in multitasking scenarios. While we acknowledge emerging studies on CMT training on younger athletes and have provided significant long-term benefits for multitasking capabilities [24–27], studies focusing on older athletes is limited. Older athletes have distinct physiological and psychological demands due to their competitive involvement over time and have built up resilience that might provide specific effect [28], highlighting the need to scope the literature to describe the effect of CMT on older athletes.

Summarily, existing literature on the effect of physical and/or cognitive rehabilitation either exists for younger athletes or older adults but is limited to older athletes. While older adults and older athletes both have the naturally occurring aging effect on their bodily system, the latter has an accumulated physiological and cognitive reserve and stamina that could be residual of previous and current competitive training. Moreover, older athletes may respond differently to training due to their unique baseline fitness levels, competitive goals, and higher engagement in systematic training compared to their non-athlete peers [28]. Therefore, therapists may treat older athletes differently during cognitive and physical rehabilitation due to their athletic background, often assuming higher baseline fitness, motivation, or resilience compared to non-athletes. This could result in therapists setting more challenging goals, providing less guidance or support, or designing rehabilitation programs that emphasize performance rather than age-related considerations [29]. Conversely, therapists might overcompensate by focusing on the athlete's age, potentially reinforcing stereotypes and underestimating their capabilities. These differing approaches could inadvertently affect the quality of care, the athlete's experience, and overall rehabilitation outcome, potentially reinforcing ageist attitudes.

Overall, older athletes’ rehabilitation needs differ from those of both older adults and younger athletes. Compared with older adults, older athletes typically have higher functional goals (e.g., return to training or competition) and may therefore require rehabilitation that targets power, rate of force development, plyometric capacity, and sport-specific skills [29–31]. Older athletes may also present with overuse or training-related problems (e.g., symptom flares) superimposed on age-related degeneration, necessitating careful load management, cross-training, and technique modification [32]. Compared with younger athletes, older athletes often demonstrate slower tissue recovery and more persistent fatigue, requiring longer recovery between heavy sessions and more conservative progression [30–32]. Rehabilitation may also need greater emphasis on maximal strength, power, balance, and neuromuscular control to reduce reinjury risk and restore performance. This underscores the importance of exploring the experiences of older athletes in cognitive and physical rehabilitation, in addition to understanding the types and effects of these intervention on several health and social outcomes. Therefore, this study aims to describe the treatment regime (frequency, intensity, time/duration, and type of volume and progression of rehab) and components of cognitive and physical rehabilitation interventions, describe their combined effects on health and social outcomes and explore older athletes’ experiences with these interventions.

## Methods

### Study design

The five-stage Arksey & O’Malley [33] framework will guide this review: (1) identifying the research question(s); (2) identifying relevant studies; (3) study selection; (4) charting the data; and (5) collating, summarizing and reporting the results. A scoping review enables researchers to understand the knowledge and research gap in a research field [33]. We choose a scoping review over a systematic review because our research question is broad, aiming to describe the effects of physical and cognitive interventions across various health, cognitive, and social outcomes. A scoping review will allow us to map key concepts such as frequency, intensity, duration, and types of rehabilitation, providing a comprehensive overview of the literature that can inform a targeted systematic review focused on specific health and social outcomes. We will report this scoping review using the Preferred Reporting Items for Systematic reviews and Meta-Analyses extension for Scoping Reviews (PRISMA-ScR) [34] – See Appendix 1. This review is registered at Open Science Framework (OSF): https://doi.org/10.17605/OSF.IO/HFT8Y. This is a scoping review and do not require ethics approval

### Stage 1: Identifying the research question(s)

The following research questions guided this review: Among older athletes, (a) what are training regime (frequency, intensity, time/duration, and type of volume and progression of rehabilitation) and components of cognitive and physical rehabilitation interventions, (b) what are the reported individual and combined effects of these interventions on health and social outcomes, and (c) what are older athletes experiences in participating in these interventions.

### Stage 2: Identifying relevant studies

The search strategy will be developed in consultation with a health science librarian. We used several MESH terms and key words following the Population (older adults) – Intervention (Physical or Cognitive Rehabilitation) – Comparison (Not Physical or Cognitive Rehabilitation) – Outcome (Health and Social Outcomes and Experiences) concepts. Incorporating outcome-related terms, such as “health outcomes” and “social outcomes,” in our preliminary MEDLINE search resulted in a high number of articles, suggesting low specificity due to the inclusion of many false positives. This was further validated by reviewing the first 100 articles, none of which met our inclusion criteria. Our health librarian advised us to remove the outcome searches, as they were redundant since interventions already include outcome measures, and we are only including experimental studies. Upon eliminating these outcome terms, the search strategy yielded a more relevant set, with five out of the first ten articles identified as potential candidates for inclusion in our study. Therefore, we have decided to remove these outcome terms from our search criteria. Therefore, our search concepts focused on Population and Intervention. We developed our search strategy using both a conceptual and objective approach [35]. The conceptual approach involves utilizing relevant concepts related to the subject matter by incorporating keywords, synonyms, and MeSH terms (when available). The objective approach typically involves using seed articles to evaluate the sensitivity of the search. We employed several seed articles, specifically Art et al. [36] for physical rehabilitation terms and Irazoki et al. [37] for cognitive rehabilitation terms, to help assess the sensitivity of our search. This ensured that our initial search strategy would yield at least one of the articles included in the seed articles. We used the search terms from these seed articles, resulting in the retrieval of three relevant articles. Conceptually, since terms related to older athletes are not comprehensive— for instance, Xiao et al's [38] search included only six terms: player, athlete, sportsman, sportswoman, sportsperson, and jock—we expanded our search by adding additional keywords and synonyms related to various sports. Each concept was combined with several MESH and keyword using OR, and later each of the concepts was combined with AND. See Table 1 for full search strategy, see our first search in Appendix 2. This search strategy will be adapted in multiple databases, including Web of Science, ProQuest, EBM Reviews, JBI EBP, Embase, APA PsychInfo, and Social Services Abstracts.

**Table 1:** Search strategy.

Concepts	
Population (Older Athletes)	(Older [MESH] OR elderly [MESH] OR geriatric [MESH] OR older people OR senior [MESH] OR retirees OR aged [MESH] OR older persons OR gerontology OR aging [MESH] OR ageing adult) adj99 (Athlete [MESH] OR Sport OR Sport person OR Sportman OR Sportwoman OR basketball player OR body builder OR boxer OR cyclist OR football player OR hockey player OR judoka OR skier OR soccer player OR triathlete OR wrestler OR golfer OR handball player OR lacrosse player OR male athlete OR female athlete OR netball player OR olympic athlete OR professional athlete OR rowers OR rugby football player OR softball player OR squash player OR table tennis player OR tennis player OR volleyball player OR water polo player)
Intervention (Cognitive and Physical Rehabilitation Interventions)	(Cognitive rehab* OR cognitive therap* [MESH] OR cognitive training [MESH] or cognitive intervention OR memory training [MESH] OR mental exercise* OR “cognitive motor training” OR “dual task” or “dual-task” OR “cognitive-motor training” OR “perceptual-training” OR brain training OR mental exercise* OR cognitive behavioral therapy OR neurofeedback [MESH] OR mindfulness [MESH] OR mental* agil* [MESH]) OR (“physical rehabilitation” OR “physical therapy” OR physiotherapy OR exercise therapy OR “physical conditioning” OR “strength training” OR “endurance training” OR “mobility training” OR “balance training” OR “proprioceptive exercises” OR “neuromuscular training” OR “physical reconditioning”)

For all databases searches, the symbol * will be used to allow the inclusion of varied word endings. Additionally, those without MESH stamps at the end of it are keywords.

### Stage 3: Selecting studies

The citation records will be uploaded into Covidence, a web-based platform designed to support systematic and advanced review processes. Duplicate entries will be identified and removed automatically. The study selection process in Covidence will include two stages: title/abstract screening and full-text screening. Both stages will begin with pilot testing. During the pilot phase, each reviewer will screen at least 100 articles for titles and abstracts.

To assess inter-reviewer reliability, we will calculate Cohen’s Kappa and the Intraclass Correlation Coefficient (ICC). If the agreement between reviewers is excellent (i.e., 0.8 or higher), the articles will be divided among the reviewers for independent screening, with weekly check-ins during research meetings to ensure accuracy. If the agreement is below 0.8 [39], two reviewers will independently review the articles, and any disagreements will be resolved by a third reviewer.

Each stage of the review process, including both title/abstract and full-text screening, will be conducted using predefined inclusion and exclusion criteria outlined below:

We will include an article if it meets the following criteria:

a) The *study population* is based on older athletes, defined as individuals aged 35 years or older, who actively participate in organized sports that emphasize regular competition and systematic training. We will include only studies that examined activities undertaken by former competitive athletes after retirement.
b) The study involves a physical or cognitive *intervention* or combination of both performed either simultaneously or sequentially. Examples of physical interventions include, but are not limited to, walking; strength training; balance or flexibility training; and sport or exercise activities such as cycling, bowling, golf, swimming, soccer, road racing, and sprint running. Similarly, examples of cognitive rehabilitation include, but are not limited to, memory strategy training, dual-task training, attention process training, and computerized cognitive training. The *intervention* must include a description of the training regime including frequency, intensity, time/duration, and type of volume and progression of rehab (FITT-VP).
c) The study must report on at least one of the following outcomes:
  - **Cognitive outcomes:** examples include but are not limited to, attention, concentration, perception, memory, comprehension, communication, executive functioning, visual spatial, reasoning, problem-solving, judgment, initiation, planning, self-monitoring, and awareness [16].
  - **Physical outcomes:** examples include but are not limited to, muscle strength, range of motion, reduced pain, balance and coordination, endurance, flexibility, recovery from sports related injuries, gait speed, blood pressure control, grip force, jump height, quality of life, proprioception, change in VO2 max, bone density, etc [14–15].
  - **Social outcomes:** examples include but are not limited to, social inclusion, communication skills, social participation, team cohesion, relationships, community integration, loneliness, depression, or anxiety associated with aging and injury, social network (quality and quantity), and social support [40].
d) All study designs will be included if:
  - **Quantitative studies:** Only experimental designs, such as randomized controlled trials (RCTs) or quasi-experimental studies, will be included.
  - **Qualitative studies:** Studies reporting the experiences of older athletes participating in cognitive or physical rehabilitation.
  - **Mixed-method studies:** Eligible if the quantitative component is experimental (RCT or quasi-experimental) and the qualitative component explores older athletes’ experiences with the intervention.

We will exclude an article if it meets any of the following conditions:

a) Quantitative studies that are non-experimental, such as cohort or cross-sectional studies, will be excluded. These study designs lack the methodological rigor to infer causality, which is critical for evaluating the effectiveness of the interventions under investigation.
b) Any study describing the development of a physical and cognitive rehabilitation program for older athletes without reporting effects on physical, cognitive, or social outcomes, or without exploring participants’ experiences.
c) Review articles will be excluded. However, the references within these reviews will be hand-searched to identify any relevant studies that meet the inclusion criteria.
d) Articles not available in English.

### Stage 4: Charting of data

The data will be charted and organised in COVIDENCE©. The relevant data to be extracted will include the following key elements:

- Study information: author name(s), publication year, country, research aim(s), study design, participant characteristics, sample size, data collection methods and data analysis type.
- Intervention – Type of intervention (physical or cognitive rehab), features of the intervention include frequency, intensity, time/duration, type of volume and progression of rehab, and type of exercise – FITT-VP.
- The outcomes for physical rehabilitation, cognitive rehabilitation, social rehabilitation, and cognitive motor training are listed above.
- Intervention effects will be extracted including effect size, pre and post mean and (SD).

Two reviewers will independently conduct a pilot data extraction exercise, after which they will convene to discuss and resolve any discrepancies. If there is persistent disagreement, a third reviewer will be consulted to provide input and facilitate consensus. See Appendix 3 – a sample of data extraction template.

### Stage 5: Collating, summarizing, and reporting the results

Study selection would be reported following PRISMA-Flowchart (see Appendix 4). We will code each study by intervention format: (1) physical-only (subtyped, e.g., strengthening, conditioning), (2) cognitive-only (subtyped, e.g., memory, processing speed), or (3) combined/multicomponent (physical + cognitive delivered concurrently or sequentially). Key metrics for physical outcomes (e.g., strength, endurance, balance), cognitive outcomes (e.g., memory, executive function), and social outcomes (e.g., social engagement, quality of life) will be extracted and summarized to provide insight into the impact of each type of intervention. For each outcome, we will extract and report effect sizes (when available) or pre–post means and standard deviations (or other summary statistics, as reported). For observational designs (e.g., cohort and cross-sectional studies), we will preferentially extract adjusted estimates (e.g., risk ratio, hazard ratio, incidence rate ratio, regression coefficient [β], odds ratio, or prevalence ratio), where provided. For combined interventions, we will report effects for the overall program and, where the study design permits (e.g., separate arms or factorial designs), we will also extract component-specific effects (physical vs cognitive) and any reported interaction/synergy. When component effects cannot be disentangled, combined interventions will be synthesized separately from single-component interventions and clearly labelled as combined-intervention effects. Results will be presented in stratified summary tables and a narrative synthesis comparing the direction and magnitude of effects across these categories.

For qualitative studies, we will apply Thomas and Harden’s [41] three-stage thematic synthesis approach: (1) line-by-line coding of the text, (2) development of descriptive themes, and (3) generation of analytic themes. Because reporting conventions vary and some disciplines present results and discussion together, our primary data will be the themes, subthemes, and participant quotations reported in the Results/Findings section. Where results and discussion are integrated, we will extract and treat participant quotations as the qualitative findings. Data will be managed and analyzed in NVivo. Verbatim qualitative findings (including participant quotations and accompanying author interpretations, where clearly presented as findings) will be imported into NVivo for analysis. In Stage 1, two reviewers will independently code the text line by line, using an inductive approach to capture meaning and content, with a focus on older athletes’ experiences of participating in physical and cognitive rehabilitation. Initial codes will be created as “free” codes (i.e., without a predefined hierarchy). In Stage 2, the two reviewers will compare their coding, identify similarities and differences, and organize codes into a hierarchical structure to develop descriptive themes. Disagreements regarding code grouping or theme development will be resolved through discussion, with input from a third reviewer as needed. In Stage 3, the review team will iteratively refine the descriptive themes into analytic themes that extend beyond the primary studies by explaining barriers, facilitators, and experiential implications relevant to participation in physical and/or cognitive rehabilitation. This process will be conducted through a series of iterative meetings (minimum of three) to ensure that the analytic themes are sufficiently abstract and account for all descriptive themes.

## Discussion

This scoping review aims to address a critical gap in the literature by synthesizing evidence on the effects of cognitive and physical rehabilitation interventions on the health and social outcomes of older athletes. While extensive research exists on cognitive and/or physical rehabilitation interventions for younger athletes and older adults, little is known about their applicability and effectiveness in older athletes. Older athletes have an accumulated physiological and cognitive reserve and stamina that could be residual of previous and current competitive training. For that reason, older athletes may respond differently to training due to their unique baseline fitness levels, competitive goals, and higher engagement in systematic training compared to their non-athlete peers [28]. Hence, collating evidence on the various interventions, has the potential to explain whether older athletes experience distinctive benefits due to their unique physiological and psychological characteristics. These benefits might manifest not only as improvements in physical outcomes (e.g., muscle strength, balance, or injury prevention) and cognitive outcomes (e.g., executive function, attention), but also in social outcomes such as team cohesion, community participation, and psychological well-being.

Importantly, the findings from this scoping review may offer practical implications for clinicians, coaches, and researchers aiming to develop or adapt rehabilitation protocols specifically for an older athletic population. Should certain interventions of physical and/or cognitive rehabilitation prove particularly advantageous, these insights can inform tailored, sport-specific rehabilitation strategies that acknowledge the baseline fitness, cognitive reserve, and competitive goals of older athletes. Moreover, gaining a deeper insight into the effects social interventions such as enhanced peer support, reduced isolation, or improved self-esteem could reinforce the broader value of sustained athletic engagement later in life. By synthesizing the existing evidence, this review can serve as a catalyst for more rigorous research, potentially guiding randomized controlled trials or mixed-methods studies that fill in the current knowledge gaps and validate the efficacy of integrated rehabilitation programs for older athletes.

### Project timeline

When writing this protocol, we are searching for each database. The estimated timeline for the project includes A) record screening (title, abstract, and full text) to be completed between Jan and Feb 2026, B) data extraction to take place from March to June 2026 and C) results anticipated by August 2026. We will also update our search, as our strategy in each database is designed to identify any new articles that may meet our inclusion criteria during the review period.

## Supporting information

Appendix 1 Preferred Reporting Items for Systematic reviews and Meta-Analyses extension for Scoping Reviews (PRISMA-ScR). (DOCX)

Appendix 2 Search Strategies. (DOCX)

Appendix 3 Data Extraction Template. (DOCX)

Appendix 4 PRISMA Flowchart. (DOCX)
