# Supplementary figures and images for: The rehabilitation experiences, individual and combined effects of cognitive and physical rehabilitation on health and social outcomes in older athletes: A scoping review protocol
Source: PLoS One. 2026 Mar 6;21(3):e0343744. doi: 10.1371/journal.pone.0343744 (PMC12965522; doi:10.1371/journal.pone.0343744)

Appendix 1 – MEDLINE Search Output


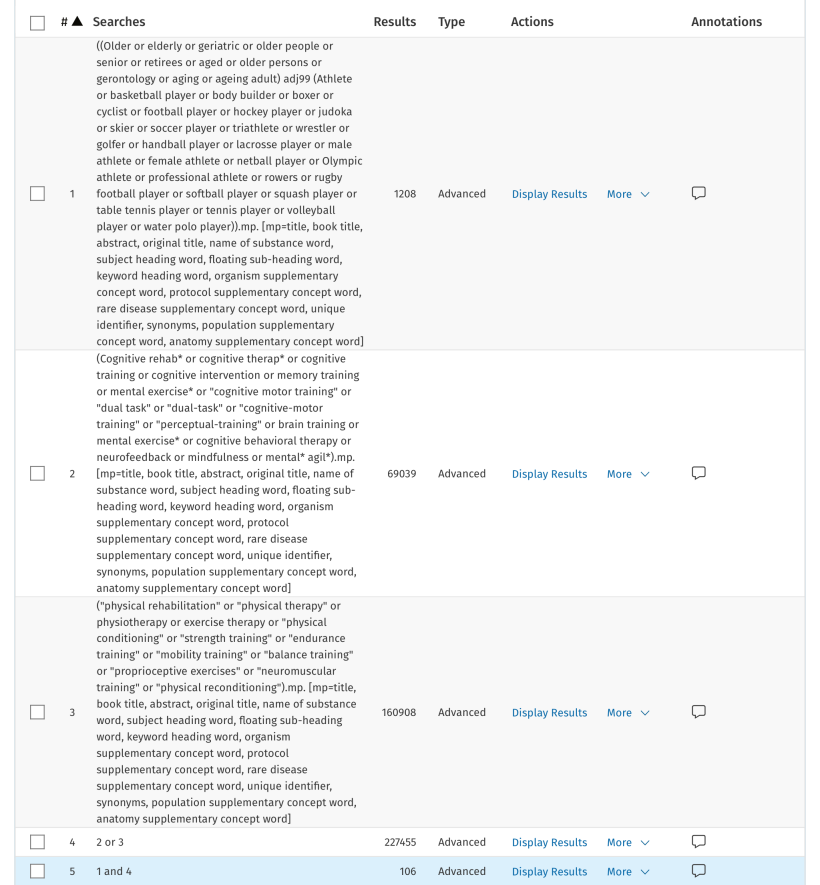

Supplement: Appendix 2 — (DOCX) [file pone.0343744.s002.docx]
