# Supplementary material for: The rehabilitation experiences, individual and combined effects of cognitive and physical rehabilitation on health and social outcomes in older athletes: A scoping review protocol
Source: PLoS One. 2026 Mar 6;21(3):e0343744. doi: 10.1371/journal.pone.0343744 (PMC12965522; doi:10.1371/journal.pone.0343744)
Supplement: Appendix 3 — (DOCX) [file pone.0343744.s003.docx]

**Appendix 3 - Data extraction Sample (Adapted from COVIDENCE©)**

# Study Meta-data

1. Study authors
2. Year of publication
3. Study country
4. Study title
5. Study aim(s)/objective(s)/hypothesis/ research question(s)

# Study Methodology

1. Research setting (community or lab etc)
2. Study design (quant, qual or mixed method)
3. Types of quantitative
4. Types of qualitative
5. Types of mixed method
6. Sampling method
7. Ethical statement present
8. Informed consent statement present
9. Data collection method
10. Data analysis

# Study Results

1. Sample size
2. % of female
3. Age (mean, SD, age range)
4. Other population characteristics – if
5. List of themes (qualitative) and quotes
6. Description of findings (quantitative)
7. If cohort or cross-sectional study, describe the exposures
8. If intervention study, describe the intervention group and control group
9. Describe the physical intervention delivered
10. Detailed description of each parameter and their reports in each study – Frequency, Intensity, Time/Duration, types of exercise, type of volume and progression
11. Description of cognitive intervention
12. Detailed description of each parameter and their reports in each study – Frequency, Intensity, Time/Duration, types of cognitive intervention, type of volume and progression
13. Physical outcomes definitions and measures, e.g strength, endurance power, balance, coordination, pain outcomes etc.
14. Description of the effect of the intervention on each measure, for example Stress ball squeezes (4 sets of 20–30 s at ~70–85% of maximal hold load), performed for 45–60 minutes per session, three sessions per week, improved handgrip strength from 20 kg to 45 kg.
15. Cognitive outcomes definitions and measures, e.g reaction time, processing speed, memory etc.
16. Description of the effect of the intervention on each measure, example as in no 11 above.

**Hint –** when describing effects, please include the effect size, pre and post mean and SD if available.

*Subject to modification during data extraction*.
